# Accurate diagnosis of apical hypertrophic cardiomyopathy using explainable advanced electrocardiogram analysis

**DOI:** 10.1093/europace/euae093

**Published:** 2024-04-08

**Authors:** Rebecca K Hughes, George D Thornton, James W Malcolmson, Iain Pierce, Shafik Khoury, Amanda Hornell, Kristopher Knott, Gabriella Captur, James C Moon, Todd T Schlegel, Martin Ugander

**Affiliations:** Institute of Cardiovascular Science, University College London, Gower Street, London, UK; Barts Heart Centre, The Cardiovascular Magnetic Resonance Imaging Unit and The Inherited Cardiovascular Diseases Unit, St Bartholomew’s Hospital, West Smithfield, London, UK; Institute of Cardiovascular Science, University College London, Gower Street, London, UK; Barts Heart Centre, The Cardiovascular Magnetic Resonance Imaging Unit and The Inherited Cardiovascular Diseases Unit, St Bartholomew’s Hospital, West Smithfield, London, UK; Barts Heart Centre, The Cardiovascular Magnetic Resonance Imaging Unit and The Inherited Cardiovascular Diseases Unit, St Bartholomew’s Hospital, West Smithfield, London, UK; William Harvey Institute, Queen Mary University of London, London, UK; Barts Heart Centre, The Cardiovascular Magnetic Resonance Imaging Unit and The Inherited Cardiovascular Diseases Unit, St Bartholomew’s Hospital, West Smithfield, London, UK; Cardiovascular Clinical and Academic Group, Molecular and Clinical Sciences Institute, St George’s University of London, London, UK; Department of Clinical Physiology, Karolinska University Hospital and Karolinska Institutet, SE-171-76, Stockholm, Sweden; Institute of Cardiovascular Science, University College London, Gower Street, London, UK; Barts Heart Centre, The Cardiovascular Magnetic Resonance Imaging Unit and The Inherited Cardiovascular Diseases Unit, St Bartholomew’s Hospital, West Smithfield, London, UK; Institute of Cardiovascular Science, University College London, Gower Street, London, UK; MRC Unit of Lifelong Health and Ageing, University College London, 1-19 Torrington Place, Fitzrovia, London, UK; Inherited Heart Muscle Conditions Clinic, Department of Cardiology, Royal Free Hospital, NHS Trust, Gower Street, London, UK; Institute of Cardiovascular Science, University College London, Gower Street, London, UK; Barts Heart Centre, The Cardiovascular Magnetic Resonance Imaging Unit and The Inherited Cardiovascular Diseases Unit, St Bartholomew’s Hospital, West Smithfield, London, UK; Department of Clinical Physiology, Karolinska University Hospital and Karolinska Institutet, SE-171-76, Stockholm, Sweden; Nicollier-Schlegel SARL, Trelex, Switzerland; Department of Clinical Physiology, Karolinska University Hospital and Karolinska Institutet, SE-171-76, Stockholm, Sweden; Kolling Institute, Royal North Shore Hospital and University of Sydney, St Leonards, Sydney, NSW 2065, Australia

**Keywords:** Electrocardiography, Vectorcardiography, Apical hypertrophic cardiomyopathy, Hypertrophic cardiomyopathy

## Abstract

**Aims:**

Typical electrocardiogram (ECG) features of apical hypertrophic cardiomyopathy (ApHCM) include tall R waves and deep or giant T-wave inversion in the precordial leads, but these features are not always present. The ECG is used as the gatekeeper to cardiac imaging for diagnosis. We tested whether explainable advanced ECG (A-ECG) could accurately diagnose ApHCM.

**Methods and results:**

Advanced ECG analysis was performed on standard resting 12-lead ECGs in patients with ApHCM [*n* = 75 overt, *n* = 32 relative (<15 mm hypertrophy); a subgroup of which underwent cardiovascular magnetic resonance (*n* = 92)], and comparator subjects (*n* = 2449), including healthy volunteers (*n* = 1672), patients with coronary artery disease (*n* = 372), left ventricular electrical remodelling (*n* = 108), ischaemic (*n* = 114) or non-ischaemic cardiomyopathy (*n* = 57), and asymmetrical septal hypertrophy HCM (*n* = 126). Multivariable logistic regression identified four A-ECG measures that together discriminated ApHCM from other diseases with high accuracy [area under the receiver operating characteristic (AUC) curve (bootstrapped 95% confidence interval) 0.982 (0.965–0.993)]. Linear discriminant analysis also diagnosed ApHCM with high accuracy [AUC 0.989 (0.986–0.991)].

**Conclusion:**

Explainable A-ECG has excellent diagnostic accuracy for ApHCM, even when the hypertrophy is relative, with A-ECG analysis providing incremental diagnostic value over imaging alone. The electrical (ECG) and anatomical (wall thickness) disease features do not completely align, suggesting that future diagnostic and management strategies may incorporate both features.

**What’s new?**

- This study explores the diagnostic power of the electrocardiogram (ECG) in apical hypertrophic cardiomyopathy (ApHCM) and demonstrates that a 12-lead ECG with advanced ECG (A-ECG) analysis can be used to reliably diagnose the disease.
- It demonstrates that ECG changes occur in the early stages of the disease, before the development of overt hypertrophy.
- The most distinguishing A-ECG features in ApHCM are not associated with maximum wall thickness, implying that more than just hypertrophy is responsible for the characteristic ECG changes.
- This allows clinicians to consider the diagnosis of ApHCM if the patient has characteristic ECG changes, even if the wall thickness criteria for HCM are not met.
- It also reminds cardiologists of the diagnostic power of a simple 12-lead ECG and to consider revisiting vectorcardiography to gain greater electrical insights.

## Introduction

The typical standard resting 12-lead electrocardiogram (ECG) in apical hypertrophic cardiomyopathy (ApHCM) has a distinctive appearance, with precordial tall R waves and deep T-wave inversion, with ‘giant’ negative T waves (>10 mm) in around half of cases.^1^ However, while frequently characteristic, such ECG changes may vary over time, and electrically milder changes can be mistaken for other phenotypes or diseases (other HCM variants, other cardiomyopathies, or myocardial ischaemia).^2^ In initial disease descriptions from Japan, ApHCM diagnostic criteria were ECG-based with the use of vectorcardiography (VCG) and with confirmation by invasive ventriculography.^3^ Advancements in imaging shifted this paradigm, and in current clinical practice, while the ECG may gatekeeper imaging diagnostic tests such as echocardiography and cardiovascular magnetic resonance (CMR), it is the imaging of apical hypertrophy that is the primary diagnostic criterion, a current de-emphasis of the electrical over anatomical phenotype components.

In left ventricular hypertrophy, the extent of secondary T-wave changes usually mirrors the amplitude of the QRS complex and R waves, and these have been shown to correlate in ApHCM,^2,4^ with giant negative T waves and R-wave voltages >25 mm having been demonstrated in those with more severe apical hypertrophy.^5^ The typical ECG appearances are also seen in those with morphologically mild ApHCM—with <15 mm apical hypertrophy but typical imaging features (loss of apical tapering, apical cavity obliteration in systole, ‘ace-of-spades’ appearance of left ventricular cavity, ±apical microaneurysms), a subgroup termed relative ApHCM.^6^ This not only calls into question the anatomical theory for the ECG changes, but also raises the question of whether the ECG changes that occur before the imaging phenotype fully develops could be relied upon for diagnosis.

The 12-lead ECG, standardized for almost 80 years,^7^ was initially supplemented by labour-intensive techniques, such as VCG, but these have retreated from mainstream clinical use with the success of cardiac imaging. However, the emergence of electronic patient records with digital raw data storage of the ECG as standard, in combination with biobanks and advanced statistical methods, permits the return of reliable insights from ECGs and their delivery at healthcare system scale. Advanced ECG (A-ECG) uses derived three-dimensional vectorcardiograms^8^ and measures of QRS- and T-wave complexity^9^ to improve the diagnostic performance of the ECG in a number of domains, but not for ApHCM to date.^10^ We sought to assess whether A-ECG analyses based on standard 12-lead ECG could accurately diagnose overt and relative ApHCM and to assess relationships between electrical and anatomical changes.

## Methods

The prospective ApHCM study was approved by the National Health Service Research Ethics Committee (NHS REC) and the Health Research Authority (HRA) and conducted in accordance with the Declaration of Helsinki. All ApHCM subjects provided written, informed consent (REC 18/LO/0188 and 17/SC/0077). Other study participants were previously recruited from various centres worldwide and provided informed consent or approval of a waiver of individual consent from a human subject ethics review board as previously described.^10^

### Study populations

Patients with suspected or confirmed ApHCM/relative ApHCM (*n* = 95) were prospectively recruited from tertiary referral cardiomyopathy clinics at St Bartholomew’s Hospital or St George’s University Hospital, London, UK, and underwent a standard resting 12-lead ECG, where digital raw data were stored electronically, and a CMR scan. Three patients were later excluded due to the presence of a complete bundle branch block. A further 15 ApHCM subjects had the same ECG assessment, but the imaging was echocardiography alone. Overt ApHCM was defined as apical maximum wall thickness (MWT) ≥ 15 mm in end diastole in conjunction with other characteristic features of the disease^11^ such as apical cavity obliteration, apical aneurysm, and suggestive ECG changes.^4^ Relative ApHCM was defined previously,^6^ as inappropriate apical hypertrophy compared with expected apical wall thickness but not exceeding 15 mm, in combination with other characteristic features of the disease, as above. The diagnosis of overt disease involved the use of CMR or echocardiography and that of relative ApHCM only CMR.

Healthy subjects (*n* = 1672) were defined as low risk, asymptomatic individuals with no cardiovascular or systemic disease, based on clinical history and physical examination. The exclusion criteria for healthy subjects included a diagnosis of and treatment for hypertension or diabetes, current smoking status, and increased blood pressure on examination (>140/90 mmHg). They were recruited from the following centres: Johnson Space Center (USA), the Universidad de los Andes (Venezuela), the University of Ljubljana (Slovenia), and Lund University Hospital (Sweden).^10^

Patients with established cardiovascular disease were subdivided into diagnostic groups: (i) occlusive coronary artery disease (*n* = 372)—the presence of ≥50% obstructed in at least one major vessel by invasive coronary angiography, or, if angiography had not been performed, the presence of one or more reversible perfusion defects in a coronary artery territory on 99m-Tc-tetrofosmin single-photon emission computed tomography, always with normal systolic function; (ii) left ventricular electrical remodelling (LVER, *n* = 108), based on the presence of at least moderate left ventricular hypertrophy (LVH) by imaging, but with normal systolic function; (iii) ischaemic and (iv) non-ischaemic cardiomyopathy (*n* = 114 and *n* = 57), with left ventricular systolic dysfunction (ejection fraction ≤50%), or (v) asymmetrical septal hypertrophy (ASH) HCM (*n* = 126). At least moderate LVH is defined using echo guidelines [left ventricular (LV) mass index as 128–145 g/m^2^ in males and 116–131 g/m^2^ in females].^12^ Wall thickness criteria are not used for the diagnosis of LVH but are part of the diagnostic criteria for hypertrophic cardiomyopathy (≥15 or ≥13 mm in the case of familial disease). Clinical acumen was used in all patients to make the ultimate diagnosis based on the presence or absence of other features of ASH HCM (e.g. systolic anterior motion of the anterior mitral valve leaflet [SAM], left ventricular outflow tract [LVOT] jet flow acceleration). These groups were identified from the following centres: Texas Heart Institute (Houston, USA), the University of Texas Medical Branch (Galveston, USA), the University of Texas Health Science Center (San Antonio, USA), Brooke Army Medical Center (San Antonia, USA), St Francis Hospital (Charleston, USA), the Universidad de los Andes (Mérida, Venezuela), and Lund Hospital (Lund, Sweden). Electrocardiograms were acquired within 30 days of the cardiac imaging examination.

### Electrocardiograms

For the 92/107 ApHCM subjects with contemporaneous CMRs, the resting 12-lead ECGs were performed on the day of CMR imaging using a Mortara ELI350 machine and stored in the patients’ electronic record (Cerner Millennium). These were extracted as DICOM files, pseudo-anonymized, and converted to the XML file format for analysis. For the remaining 15 subjects, 5 min ECGs were performed. The other disease/control groups had a combination of 5 min and 10 s ECGs. There were three analyses: First, a visual assessment of ECG DICOMs seeking the typical 12-lead ECG features of ApHCM—specifically tall R waves [precordial leads (V1–V6) ≥ 14 mm] and associated T-wave inversion (≥3 mm). Secondly, conventional ECG measures of scalar durations, amplitudes included the following: 12-lead voltage, Cornell voltage (the sum of the S wave in V3 plus the R wave in lead aVL, where ECG LVH is defined as >2.8 mV for males and >2.0 mV for females),^13^ and Sokolow–Lyon criteria (the sum of the S wave in V1 plus the larger of the R wave in V5 or V6, where ECG LVH is defined as >3.5 mV).^10,14^ Thirdly, A-ECG analysis involved derived VCG and polarcardiographic measures of the planar and three-dimensional spatial angles, directions (azimuths and elevations), and magnitudes of the electrical activation pattern, and QRS- and T-wave complexity measures quantified by singular value decomposition (SVD).

### Cardiovascular magnetic resonance acquisition and analysis

Apical hypertrophic cardiomyopathy subjects underwent CMR including mapping and late gadolinium enhancement (LGE) to exclude phenocopies. Scans were performed at the Barts Heart Centre and the Chenies Mews Imaging Centre on a 1.5 T magnet (Aera, Siemens Healthcare, Erlangen, Germany) using standard clinical protocols. Cardiovascular magnetic resonances were analysed using commercially available software (CVI42, Circle Cardiovascular Imaging, Calgary, Canada). Left ventricular volume analyses used a validated machine-learning algorithm,^15^ as did MWT.^16^ Late gadolinium enhancement was quantified using the full-width half-maximum technique, with LGE expressed in grams and as a percentage of total myocardium. An apical aneurysm (≥5 mm) or microaneurysm (<5 mm) was defined by the presence of an akinetic/dyskinetic motion, scarring, and a non-obliterating apical cavity typically distal to an area of obliteration.

### Statistical analysis

Statistical analysis was performed using SAS JMP 11.0 (Cary, NC, USA) and R version 4.1.2 (R Foundation for Statistical Computing, Vienna, Austria) with packages MASS for linear discriminant analysis (LDA) and multiROC for receiver operator curve analysis. Normality was assessed visually on histograms and using the Kolmogorov–Smirnov test. Normally distributed and non-normally distributed continuous data were presented as mean ± standard deviation or median (interquartile range), respectively, and compared across participant groups using the independent Student’s *t*-test or Mann–Whitney–Wilcoxon test, as appropriate. Categorical data were presented as counts and percentages and compared using the *χ*^2^ test. Correlation was assessed with Pearson’s or Spearman’s coefficient for normally and non-normally distributed data, respectively. A *P*-value <0.05 was considered statistically significant. Multivariable logistic regression analysis was performed to see which A-ECG measures, when combined, differentiated ApHCM from all other patients, either diseased or healthy. Measures demonstrating significant differences by univariate analyses were then subjected to multivariable logistic regression analyses by using standard stepwise procedures. Linear discriminant analysis was also performed to test the accuracy of distinguishing each disease, including ApHCM, from every other disease and from health through similar A-ECG-related feature selection. To determine the diagnostic accuracy of both the logistic regression and the LDA, the areas under the receiver operating characteristic (AUC) curve were calculated, and bootstrap resampling was performed for a total of 3000 times to estimate prospective performance and to obtain 95% confidence intervals (CIs) for sensitivity, specificity, and AUC.

## Results

A total of 107 subjects with confirmed overt or relative ApHCM had a 12-lead ECG available for A-ECG analysis. Of these, 92/107 had contemporaneous CMR. Sixty of the 92 (65%) were classified as overt ApHCM [apical MWT ≥15 mm, age 58 ± 13 years, body surface area (BSA) 1.92 ± 0.20 m^2^, 75% male] and 32/92 (35%) as relative ApHCM (apical MWT <15 mm but other characteristic features of the disease, as described above, age 56 ± 14 years, BSA 1.88 ± 0.13 m^2^, 78% male). The demographic, baseline ECG, and CMR characteristics of the ApHCM subjects are detailed in *Table 1*.

**Table 1:** Comparison of baseline demographics, standard ECG characteristics, and CMR characteristics of relative vs. overt ApHCM

	All ApHCM	Relative ApHCM	Overt ApHCM	*P*-value (relative vs. overt)
Number of subjects, *n* (%)	92 (100)	32 (35)	60 (65)	n/a
Age, years	58 ± 13	56 ± 14	58 ± 13	0.48
BSA, m^2^	1.93 ± 0.21	1.88 ± 0.13	1.92 ± 0.2	0.28
Male subjects, *n* (%)	70 (76)	25 (78)	45 (75)	0.74
Cornell voltage, mV	1.7 (1.2–2.0)	1.5 (1.1–2.1)	1.6 (1.0–2.0)	0.73
Sokolow–Lyon, mV	3.2 (2.5–4.2)	3.7 (2.9–4.5)	3.2 (2.5–4.1)	0.18
12-Lead voltage, mV	19.7 (17.0–24.5)	20.3 (18.1–24.8)	19.0 (16.6–24.5)	0.46
Presence of giant negative T waves, *n* (%)	23 (23)	7 (22)	16 (27)	0.61
Presence of aneurysm/microaneurysm (%)	28 (30)	3 (9)	25 (42)	**<0.005**
LA area index, mm/m^2^	14 (12–16)	13 (12–16)	14 (12–17)	0.38
LVEDVi, mL/m^2^	72.8 ± 13	74.0 ± 13	72.1 ± 14	0.48
LVESVi, mL/m^2^	16 (13–20)	16 (14–22)	16 (12–19)	0.26
LVEF, %	78 (73–82)	77 (70–81)	78 (74–83)	0.36
LVSV, mL	112 ± 26	111 ± 24	112 ± 28	0.77
LV mass index, g/m^2^	77 (63–98)	65 (57–71)	88 (72–103)	**<0**.**001**
LV MWT, mm	18 ± 5	12 ± 2	20 ± 3	**<0**.**001**
T1, ms	1036 ± 36	1010 ± 33	1049 ± 31	**<0**.**001**
LGE, g	14 (0–30)	0 (0–4)	24 (14–34)	**<0**.**001**
LGE, % of LV	12 (0–22)	0 (0–4)	18 (10–24)	**<0**.**001**

Values in bold denotes statistical significance. ApHCM, apical hypertrophic cardiomyopathy; BSA, bovine serum albumin; CMR, cardiac magnetic resonance; ECG, electrocardiogram; LA, left atrium; LVEDVi, indexed left ventricular end-diastolic volume; LVEF, left ventricular ejection fraction; LVESVi, indexed left ventricular end systolic volume; LGE, late gadolinium enhancement; LV, left ventricular; LVSV, left ventricular stroke volume; MWT, maximum wall thickness.

### Amplitude criteria

**Figure 1:**
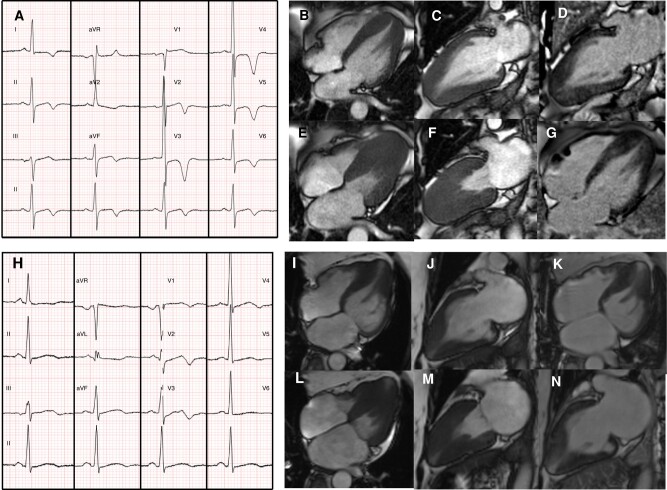
Typical vs. atypical ECG features in two patients with apical hypertrophic cardiomyopathy with corresponding CMR imaging. Electrocardiogram demonstrating visually typical ECG changes for ApHCM (*A*). Corresponding CMR demonstrates end-diastolic four-chamber (*B*) and two-chamber (*C*) views, showing typical apical hypertrophy. End-systolic four-chamber (*E*) and two-chamber (*F*) views demonstrate significant apical cavity systolic obliteration. Patchy areas of scar are shown in LGE views (*D*, *G*). In contrast, atypical ApHCM ECG appearances are seen in *H*, with CMR demonstration of apical hypertrophy (*I*, *J*) in end diastole, with the presence of an apical aneurysm in end systole (*L*, *M*). Minimal apical scar seen on LGE views (*K*, *N*). ApHCM, apical hypertrophic cardiomyopathy; CMR, cardiac magnetic resonance; ECG, electrocardiogram; LGE, late gadolinium enhancement.

Visual ECG assessment was performed on the 92 ApHCM subjects with contemporaneous CMRs. Seventy-eight of the 92 (85%) subjects had typical ECG features by the amplitude criteria of ApHCM. Of the remaining 14 subjects with atypical ECG features, 5/14 had relative ApHCM and 9/14 overt disease by CMR. Four of the 14 had apical aneurysms/microaneurysms and 10/14 did not. Fifty-three of the 78 (68%) of those with typical ECG changes had an apical scar with a total scar burden of 13% (0–22%) of the left ventricle. Nine of the 14 (64%) of those with atypical ECG features had an apical scar and a total scar burden of 9% (0–21%) of the left ventricle (*P* = 0.76). *Figure 1* shows a comparison of ECGs in patients with ApHCM with typical and atypical ECGs, with corresponding CMR images.

### Advanced electrocardiogram features

Multiple ECG variables differed between individuals with ApHCM vs. those without in univariable analysis. However, multivariable analysis revealed that the following four ECG measures, when combined, had the best ability to distinguish ApHCM from all other pathologies and from health: (i) the direction of the peak of the T-wave loop in the VCG horizontal plane; (ii) the spatial peaks QRS-T angle; (iii) the natural logarithm of the amplitude of the second eigenvector of the T wave after SVD; and (iv) the depth of the Q wave in derived VCG lead Z (*Table 2*). The AUC (bootstrapped 95% CI) of the multivariable logistic regression score incorporating these four measures was 0.982 (0.965–0.993; *Table 3*).

**Table 2:** Advanced ECG measurements in ApHCM compared with all other healthy or diseased groups combined, including *P*-values from the logistic regression score for their discrimination

ECG variable	ApHCM	All other groups combined	*P*-value
Spatial peaks QRS-T angle (degrees)	154 (137–161)	27 (16–45)	<0.001
Direction of the peak of the T-wave loop in the derived VCG horizontal plane (sine radians)	−0.26 (−0.53 to 0.06)	0.53 (0.34–0.70)	<0.001
Amplitude of second singular value of the T-wave (µV)	99 (68–162)	80 (55–111)	<0.001
Amplitude of the Q wave in derived VCG lead Z (µV)	−5343 (−862 to −347)	−280 (−426 to −164)	<0.001

Data shown as median (interquartile range).

ApHCM, apical hypertrophic cardiomyopathy; ECG, electrocardiogram; VCG, vectorcardiographic.

**Table 3:** Strength of association (χ^2^) and beta coefficient for calculating a multivariable logistic regression score for optimally differentiating between ApHCM and all other diagnosis groups, whether healthy or diseased

ECG variable	Chi-squared	Βeta coefficient	*P*-value
Intercept	–	−16.171	<0.001
Spatial peaks QRS-T angle (degrees)	110	0.045	<0.001
Direction of the peak of the T-wave loop in the derived VCG horizontal plane (sine radians)	75	−2.619	<0.001
Amplitude of second singular value of the T-wave after SVD (µV)	16	1.176	<0.001
Amplitude of the Q wave in derived VCG lead Z (µV)	14	−0.002	<0.001

ApHCM, apical hypertrophic cardiomyopathy; ECG, electrocardiogram; SVD, singular value decomposition; VCG, vectorcardiographic.

### Linear discriminant analysis performance

**Figure 2:**
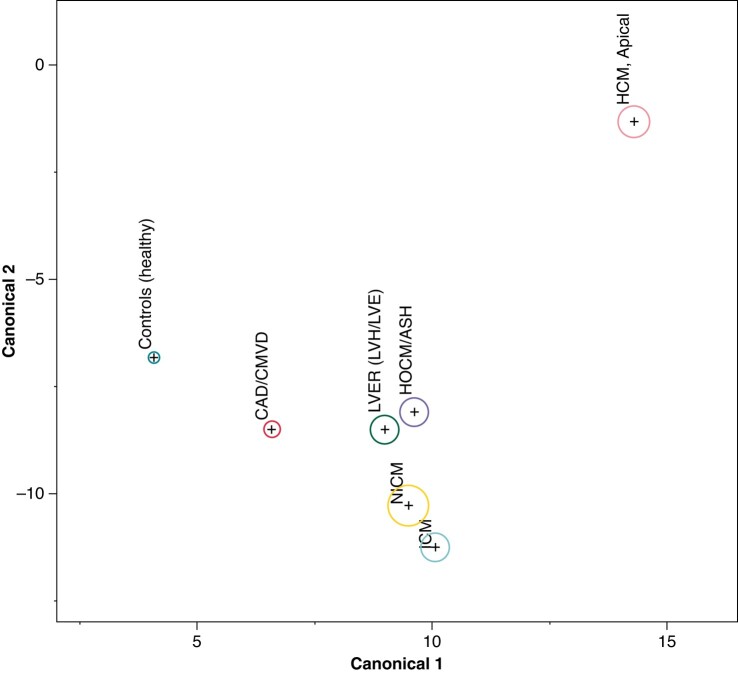
Linear discriminant analysis projection of ApHCM diagnosis. Canonical plot demonstrating the clear distinction of ApHCM from the other disease groups and from health by LDA. The circle size represents the 95% CI for the mean location of the given group. Note how this plot illustrates the distinctness of ECG characteristics for different diagnoses. ApHCM, apical hypertrophic cardiomyopathy; CAD/CMVD, coronary artery disease/coronary microvascular disease; CI, confidence interval; ECG, electrocardiogram; ICM, ischaemic cardiomyopathy; HCM, apical, apical hypertrophic cardiomyopathy; HCM/ASH, asymmetrical septal hypertrophic cardiomyopathy; LDA, linear discriminant analysis; LVER (LVH/LVE), left ventricular electrical remodelling; NICM, non-ischaemic cardiomyopathy.

Linear discriminant analysis was also performed to determine not only the extent to which A-ECG could distinguish ApHCM from other disease conditions, but also the other conditions from one another. Thirty parameters, all with both univariate and final model-related individual *P*-values of <0.001, were included in a final LDA model, with an overall accuracy of 91.6% (2342/2556), sensitivity of 88.8%, and specificity of 99.3%, AUC of 0.989 (0.986–0.991; *Table 4*, *Figure 2*). Overall LDA performances for separating the other disease conditions from one another and from cardiac health are detailed in *Table 5*.

**Table 4:** Diagnostic performance of LDA for predicting the respective ground truth diagnoses

		LDA prediction						
		Healthy	CAD	LVER	ICM	NICM	ASH HCM	ApHCM
Ground truth disease state	Healthy	1664	8	0	0	0	0	0
	CAD	38	322	6	3	1	1	1
	LVER	5	17	69	4	1	10	2
	ICM	0	4	4	87	13	3	3
	NICM	1	4	4	7	35	4	2
	ASH HCM	15	10	14	5	2	70	10
	ApHCM	0	1	4	4	0	3	95

The numbers denote numbers of individuals out of the full population (*n* = 2451).

ApHCM, apical hypertrophic cardiomyopathy; ASH HCM, asymmetrical septal hypertrophy hypertrophic cardiomyopathy; CAD, coronary artery disease; ICM, ischaemic cardiomyopathy; LDA, linear discriminant analysis; LVER, left ventricular electrical remodelling; NICM, non-ischaemic cardiomyopathy.

**Table 5:** Linear discriminant analysis performance for identifying the respective diseases

Disease group	Sensitivity (%)	Specificity (%)	Positive likelihood ratio	Inverse negative likelihood ratio	AUC (95% CI)
Healthy	98.7	98.9	89.7	76.9	0.997 (0.996–0.998)
Coronary artery disease	87.8	97.8	39.9	8.0	0.988 (0.984–0.991)
Left ventricular electrical remodelling	67.6	98.4	42.3	3.0	0.965 (0.941–0.981)
Ischaemic cardiomyopathy	79.1	98.9	71.9	4.7	0.989 (0.984–0.993)
Non-ischaemic cardiomyopathy	67.3	99.1	74.8	3.0	0.972 (0.951–0.988)
Asymmetrical septal hypertrophic cardiomyopathy	76.9	97.7	33.4	4.2	0.946 (0.915–0.970)
Apical hypertrophic cardiomyopathy	89.1	99.2	111.4	9.1	0.985 (0.963–0.996)

The 95% CIs were derived from the bootstrapping procedure described in the Statistical analysis section.

AUC, area under the receiver operating characteristic; CI, confidence interval.

### Linear discriminant analysis misclassification

Twelve subjects with ApHCM (six relative and six overt) were misclassified as another diagnosis, namely: coronary artery disease in one subject with a large apical aneurysm, ischaemic cardiomyopathy in four subjects (one with ApHCM and an apical microaneurysm, one with ApHCM and an apical aneurysm, and two with relative ApHCM), LVER in four subjects (three with relative ApHCM and one with ApHCM), and ASH HCM in three subjects (one with mixed septal and apical disease, one with ApHCM and apical aneurysm, and one with relative ApHCM). No subjects with ApHCM were misclassified as healthy. Eleven of the 12 of these subjects had visually atypical resting 12-lead ECGs by our proposed amplitude criteria. The subject with typical visual ECG appearances (with a maximum R-wave amplitude of 25 mm and maximum T-wave depth 7 mm) had overt ApHCM with an MWT 22 mm and significant apical LGE and was misclassified as ischaemic cardiomyopathy. This patient also had a contemporaneous coronary computerized tomography angiogram which showed only mild, non-obstructive coronary artery disease.

Of the remaining 2449 subjects, comprising the other healthy or disease groups studied in the LDA, 19 were misclassified as having ApHCM (0.8%). Of these, 1 had coronary artery disease, 2 had LVER, 3 had ischaemic cardiomyopathy, 2 had non-ischaemic cardiomyopathy, and 11 had ASH HCM (all of whom had a degree of apical hypertrophy). No healthy volunteers were misclassified as having ApHCM.

### Vectorcardiographic features of apical hypertrophic cardiomyopathy

Within both the logistic regression and the LDA, the direction of the peak of the T-wave loop in the derived VCG horizontal (transverse) plane and the spatial peaks QRS-T angle were the most discriminative variables. In ApHCM, the QRS loop is typically directionally normal, but with increased QRS voltages, anecdotally suggested to manifest in increased Sokolow–Lyon voltage on the conventional ECG.^17^ The main driver of increased spatial peaks QRS-T angle in ApHCM is, therefore, an abnormally directed T-wave loop, with abnormal rightward displacement of the T loop in the frontal and horizontal planes, and abnormal posterior displacement in the left sagittal plane.

**Figure 3:**
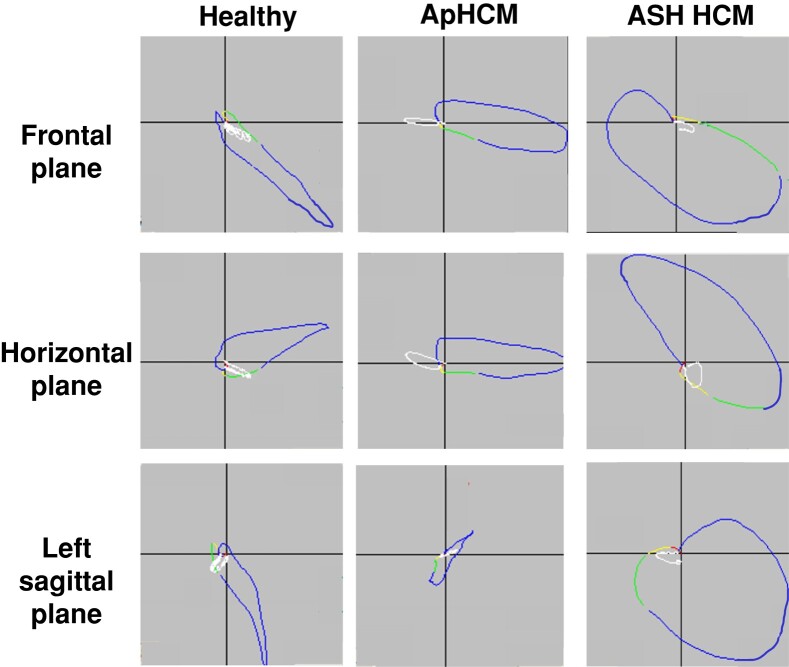
Vectorcardiographic appearances. Representative VCG loops showing differences between the VCG appearances of a single healthy subject (far left column) vs. a patient with ApHCM (middle column) and a patient with ASH HCM (far right column). In healthy subjects, there is normal orientation of the QRS loops (in blue, with the initial direction of the loops shown in yellow and then green) and T-wave loops (in white). In ApHCM, there is typically a grossly abnormal rightward direction of the T-wave loops in the frontal and horizontal (transverse) planes and an abnormal posterior direction of the T-wave loop in the left sagittal plane. These severe T-wave directional changes result in notably increased spatial peak QRS-T angles, even though QRS loops are mostly normally directed. In contrast, in ASH HCM compared with ApHCM, the T-wave loops are less abnormally directed in all planes, generally resulting in less abnormal spatial peaks QRS-T angles, although the T-wave loops are usually still directed abnormally anteriorly in the horizontal and left sagittal planes. In contrast, the QRS loops in ASH HCM are more abnormal than in ApHCM, often with the telltale excessive posterior deflection of the late portion of the QRS loop in the horizontal and left sagittal planes. ApHCM, apical hypertrophic cardiomyopathy; ASH, asymmetrical septal hypertrophy; VCG, vectorcardiographic.

By comparison, in the current study, ASH HCM more often had abnormally directed QRS loops, especially excessively posteriorly directed QRS loops in the horizontal plane, a reflection of pathological LVER. This finding is often also accompanied by an initial rightward septal delay at 30 ms into the QRS loop, and with Cornell QRS voltages typically being more abnormally high than Sokolow–Lyon QRS voltages. The T-wave loops in ASH HCM were usually less abnormally directed than in ApHCM, often being only modestly more anterior than normal in the horizontal plane, and rarely as overtly rightward as those in ApHCM (*Figure 3*). Furthermore, for purposes of comparison with previous ECG findings in ApHCM,^2^ the R-wave amplitude in V5 was shown to correlate with T-wave depth in V5 in patients with ApHCM in this study (*r* = −0.72, *P* < 0.001).

### Comparison of relative and overt apical hypertrophic cardiomyopathy

To assess whether electrical differences exist between those who reach current anatomical diagnostic thresholds for ApHCM compared with those with relative ApHCM who do not, univariable analysis was used to investigate if any of the ECG variables within the LDA predicted LV MWT (see Supplementary material online, *Table S1*). None of the variables did.

### Apical aneurysms

Twenty-eight of the 92 (30%) ApHCM subjects had apical aneurysms or microaneurysms; with a similar proportion of those with relative (3/32; 9%) vs. overt (9/60; 15%) ApHCM. Scar burden was greater in those with aneurysms vs. without [23% (16–29) vs. 6% (0–17), *P* = 0.001]. Four of the 28 (14%) had ECGs misclassified by the LDA (3 as having ischaemic cardiomyopathy, 1 as having ASH HCM; 2 with true aneurysms, and 2 with microaneurysms) vs. 8/67 (12%) ApHCM subjects without aneurysms (*P* = 0.75).

### Late gadolinium enhancement

There was no difference in overall LGE scar burden, measured as a percentage of LV myocardium, in those patients with ApHCM who were accurately diagnosed by the LDA vs. those misdiagnosed [12.6% (0–22.6) vs. 7.2% (0–18.8), *P* = 0.43]. Furthermore, an additional univariable analysis was performed to investigate if any of the variables used in the optimal logistic regression equation that distinguished ApHCM from other diseases could predict LGE, and none did (see Supplementary material online, *Table S2*).

## Discussion

The main finding of the study is that ApHCM can be reliably and accurately diagnosed by a standard 12-lead ECG with A-ECG analysis. Although further cardiac imaging has a natural role in risk stratification, the standard ECG alone can be used to diagnose ApHCM, even in those with typical imaging features but <15 mm apical hypertrophy currently required for diagnosis (relative ApHCM). This is important, since currently those with suggestive ECG findings but <15 mm apical hypertrophy are missing out on an important diagnosis and subsequent management due to not fulfilling imaging-based wall thickness criteria. However, with the presence of disease convincingly demonstrated by A-ECG, it rather suggests that this is an early or mild phenotype that should not be ignored. This argument is strengthened by the logistic regression analysis results, demonstrating that none of the four A-ECG measures that together best distinguished ApHCM from other diseases had an association with MWT. This highlights that electrical and anatomical features do not always align, and that the distinctive ECG features in ApHCM are not solely due to macroscopic apical hypertrophy.

As has been shown by others previously, we found a strong correlation between R-wave amplitude and T-wave depth. Tall R waves in ApHCM were hypothesized to be due to the anatomical location of hypertrophy causing unopposed expression of depolarization vectorial forces.^4^ However, myocardial mass and diffuse myocardial fibrosis, quantifiable using CMR, have been shown to have independent and opposing effects upon ECG voltage measures of LVH.^18^ Similarly, according to that view, T-wave changes would therefore be secondary, usually mirroring in extent the amplitude of QRS complexes and R waves, as in other forms of ventricular hypertrophy. Therefore, it should be expected that large R waves are associated with large secondary T-wave changes with opposite vectorial orientations to the cardiac apex (superiorly, rightwards, and posteriorly).^4^ The strong correlation between R-wave amplitude and T-wave depth suggests that the two are indeed interlinked. However, given the poor association between the diagnostically most predictive A-ECG measures and wall thickness, there are likely other pathophysiological processes responsible for the ECG changes than previously thought. This is supported by findings that wall thickness alone could not explain T-wave inversion in those with ApHCM with septal involvement.^19^ The authors hypothesized that ionic remodelling in ApHCM results in longer action potential duration, delayed repolarization, and inverted T waves, but found that ionic remodelling had no effect on the QRS complex in the same group. However, this explanation offers little to explain the tall R waves.

Advanced ECG performance was similar for those patients with ApHCM with and without apical aneurysms. Although this may be due to small sample numbers, it could also reflect the broad phenotypic changes encompassed by the term ‘aneurysm’. Those with large apical aneurysms tend to have a transmural scar within the aneurysm, with little or no myocardium contributing electrically; therefore, the overall electric signal may not conduct too differently. Apical cavity systolic obliteration is a key feature of the disease and, in those with aneurysms, occurs above the level of the aneurysm, which may also affect the electrical signalling. Furthermore, late gadolinium had no bearing on the accuracy of the LDA and did not predict any key A-ECG features that best distinguished ApHCM from other diseases. This is in line with other work using ECG to predict LGE in the left bundle branch block, which showed that ECG parameters predicted scars with poor accuracy.^20^ Understanding how A-ECG features change with the clinical course of the disease will be key to future disease progression tracking and may have prognostic implications. More work is needed here.

The LDA performed with high accuracy and had further diagnostic uses beyond its ability to diagnose ApHCM. Specifically, it distinguished all forms of heart disease from one another, and from health, with very good diagnostic accuracy. Importantly, in those in whom the LDA misclassified ApHCM into another group, no patient was misclassified as being healthy. Typical ECG features defined as the proposed ‘amplitude criteria’ on visual read were present in 85% of subjects, and the LDA accurately identified ApHCM in 89% of subjects. Although these proportions are similar, A-ECG is still advantageous as visual ECG assessment is not diagnostic for ApHCM, and the proposed LDA results have demonstrated excellent diagnostic capabilities. Advanced ECG also offers a quantitative characterization of the ECG findings in ApHCM relative to other pathologies, with results presented as a continuum of likelihood ranging from 0 to 100%.

The LDA performed better for ApHCM than for ASH HCM. We propose this to be a result of the imaging and electrical phenotype in ApHCM being far more homogeneous than in ASH HCM. The extent and location of hypertrophy in ASH HCM can be quite different between patients, which is likely to be reflected in the ECG changes. We do not have genetic information available for ASH HCM or ApHCM groups. However, the ASH HCM group has (i) somewhat heterogeneous imaging findings, (ii) a higher comparative prevalence of sarcomere gene mutations, and (iii) a relatively greater established knowledge base regarding the fact that genotype-positive and genotype-negative patients can have different imaging features, particularly in the context of LGE. Taken together, this may also reflect why the A-ECG performs comparatively worse in differentiating ASH HCM from ApHCM.

With large biobanks of scans and ECGs now widely available, A-ECG technology will enable us to develop our understanding of and continue to refine the diagnosis of cardiac disease, and health in the future using a simple 12-lead ECG. This may be advantageous in clinical practice by reliably speeding up diagnosis and guiding further investigations, if embedded into the clinical workflow. Although hospital care has near-immediate access to diagnostic tests, this is not always the case in the primary care setting. A reliable diagnosis from a standard ECG could help expedite appropriate management and referrals. This also has potential use in diagnostic grey areas, for example, distinguishing hypertensive heart disease from HCM, or athletic cardiac remodelling from dilated cardiomyopathy, whereby an A-ECG diagnosis would provide confidence to aid appropriate management. Furthermore, invasive electrophysiological studies have been shown to be superior to standard risk prediction models at identifying those at risk of fatal arrhythmias in HCM.^21^ Similarly, measuring the QRS peak on a 3 min standard ECG to assess the QRS fragmentation is a metric that has been shown to predict ventricular arrhythmias in those with HCM, and in combination with scar quantification, offers improved risk stratification.^22^ Further work is needed to understand whether A-ECG may have the power to identify those most at risk of sudden cardiac death in this disease cohort.

### Limitations

The data set was not divided into training and test sets, but instead cross-validated through bootstrap resampling. Although such resampling allows for the estimation of prospective accuracy within reasonable confidence limits, fully prospective validation is ultimately required. Moreover, the diagnosis of ApHCM by A-ECG has also not yet been determined to confer any additional prognostic information, and such studies are justified. Now that the diagnostic performance has been determined, this forms the foundation for subsequent studies of prognosis and management. The number of relative patients with APHCM and patients with apical aneurysms was small, and further training of the model with more data from each of these subgroups might be needed to optimize consistency of classification. Cardiovascular magnetic resonance data were available only for the ApHCM cohort; therefore, imaging comparisons between the disease populations could not be made.

## Conclusions

Apical hypertrophic cardiomyopathy is as much an electrical as an imaging phenotype and can be accurately diagnosed using a standard 12-lead ECG with A-ECG analysis even in those with typical ECG features but less than the current 15 mm apical hypertrophy required for diagnosis. Electrical (ECG) and anatomical (wall thickness) findings do not necessarily align, and macroscopic apical hypertrophy alone is not wholly responsible for ECG changes and thus should not be relied upon in isolation for diagnosis and management.

## Supplementary Material

euae093_Supplementary_Data
