# Supplementary material for: Accurate diagnosis of apical hypertrophic cardiomyopathy using explainable advanced electrocardiogram analysis
Source: Europace. 2024 Apr 8;26(4):euae093. doi: 10.1093/europace/euae093 (PMC11057018; doi:10.1093/europace/euae093)
Supplement: euae093_Supplementary_Data [file euae093_supplementary_data.docx]

**Supplemental Table 1.** Univariate predictors of maximum wall thickness using the 30 A-ECG parameters entered into the linear discriminant analysis model.

|  | r | P value |
| --- | --- | --- |
| QRS axis (⸰) | -0.143 | 0.177 |
| Angle between the first eigenvector (e1) of the QRS and the first eigenvector of the T after performing SVD of the signal-averaged QRS and T waves from the 3 derived vectorcardiographic lead channels rather than from the 8 independent channels of the scalar 12-lead ECG (⸰) | -0.236 | **0.024** |
| Age (years) | 0.012 | 0.909 |
| **Spatial peaks QRS-T angle (⸰). In the derived vectorcardiogram, the angle in three-dimensional space between the peak magnitude of the ´QRS loop and the peak magnitude of the T loop, measured in degrees.** | 0.160 | 0.129 |
| Spatial mean QRS-T angle (⸰). In the derived vectorcardiogram, the angle in three-dimensional space between the mean direction of the QRS loop and the mean direction of the T loop, measured in degrees. | 0.049 | 0.646 |
| Spatial ventricular activation time, also referred to as spatial intrinsicoid deflection. The time from the beginning of the spatial QRS wave to the peak of the R wave of the same spatial QRS wave (ms) | 0.396 | **<0.001** |
| QRS interval as derived from the vector magnitude (vectorcardiographic sum) of the Frank X, Y and Z leads (ms) | 0.217 | **0.039** |
| Time-voltage of the spatial mean T wave (mV*ms) | 0.188 | 0.075 |
| Elevation angle of the spatial ventricular gradient as projected into the frontal plane (⸰) | 0.033 | 0.756 |
| Natural logarithm of the amplitude of the third eigenvector of the T-wave, after SVD of the signal-averaged T wave (Ln µV) | 0.041 | 0.703 |
| Heart-rate corrected spatial JT interval (ms) | 0.277 | **0.008** |
| Normalized amplitude of the second eigenvector of the T-wave, after SVD of the signal-averaged T wave (µV) | 0.015 | 0.886 |
| Normalized amplitude of the second eigenvector of the QRS wave, after SVD of the signal-averaged QRS wave (µV) | -0.056 | 0.600 |
| T-wave dipolar voltage (Ln µV) | 0.132 | 0.213 |
| Duration of the R wave in the Frank Z-lead (ms) | 0.127 | 0.231 |
| Percentage of the total QRS loop area in the left sagittal plane that subtends the right lower (=posterior inferior) quadrant of that left sagittal plan (%) | 0.133 | 0.210 |
| Amplitude of the R wave in lead Y (µV) | -0.011 | 0.916 |
| **Amplitude of the Q wave in lead Z (µV)** | 0.197 | 0.061 |
| Maximum amplitude of the 3-dimensional QRS loop (µV) | 0.186 | 0.077 |
| Direction which the QRS loop in the frontal plane points when that planar loop's voltage is at a maximum (⸰) | 0.177 | 0.094 |
| Direction which the QRS loop in the sagittal plane points when that planar loop's voltage is at a maximum (⸰) | 0.057 | 0.593 |
| Azimuth angle of the 3-dimensional QRS loop when two eighths of the way into the loop (⸰) | -0.278 | **0.008** |
| Natural logarithm of the normalized QRS non-dipolar voltage (Ln µV) | 0.257 | **0.014** |
| QRS-T angle in the frontal plane (⸰) | 0.171 | 0.106 |
| QRS-T angle in the horizontal plane (⸰) | 0.049 | 0.648 |
| Ratio between the natural logs of the QRS and T non-dipolar voltage sums, after SVD | 0.160 | 0.131 |
| Maximum QRS-T angle within the three individual vectorcardiographic planes, collectively (⸰) | 0.025 | 0.817 |
| Absolute value of the QRS voltage (R peaks to S troughs or Q troughs to R peaks, whichever applies in each given lead, summed over all 12 conventional ECG leads (µV) | 0.061 | 0.564 |
| Azimuth angle, converted to sine radians, of the spatial ventricular gradient in the sagittal plane (ms) | 0.185 | 0.080 |
| Azimuth angle, converted to sine radians, of the spatial ventricular gradient in the horizontal plane (ms) | -0.016 | 0.83 |
| **Maximum T-wave angle in horizontal plane, converted to sine radians (ms)** | -0.034 | 0.747 |
| Azimuth angle in sine radians of the QRS loop when three eighths of the way into the loop) | -0.192 | 0.068 |
| Ventricular gradient [VG, vectorial sum of spatial QRS and spatial T integral vectors] optimised for right ventricular pressure overload [VG magnitude at elevation 27 degrees and azimuth 155 degrees]^23^ (mV*ms) | 0.002 | 0.985 |
| Spatial mean T wave minus spatial mean QRS^24^ (mV*ms) | 0.299 | **<0.005** |
| Arithmetic subtraction of the spatial mean T-wave from the spatial ventricular gradient (mV*ms) | -0.031 | 0.774 |
| Vectorcardiographic maximum amplitude of the T-wave divided by mean amplitude of the T-wave in the vectorcardiogram (unitless) | 0.128 | 0.228 |
| P wave duration (ms) | -0.079 | 0.474 |
| Maximum T-wave angle in the sagittal plane, converted to sine radians (ms) | -0.014 | 0.897 |
| **Natural logarithm of the amplitude of the second eigenvector of the T-wave, after SVD of the signal-averaged T wave (ln µV)** | -0.018 | 0.879 |

**Supplemental Table 2.** Univariate predictors of percentage of late gadolinium enhancement using the 4 A-ECG parameters used in the logistic regression equation.

|  | r | P value |
| --- | --- | --- |
| **Spatial peaks QRS-T angle (⸰). In the derived vectorcardiogram, the angle in three-dimensional space between the peak magnitude of the QRS loop and the peak magnitude of the T loop, measured in degrees.** | 0.03 | 0.81 |
| **Maximum T-wave angle in horizontal plane in degrees, converted to sine radians** | 0.06 | 0.59 |
| **Natural logarithm of the amplitude of the second eigenvector of the T-wave, after SVD of the signal-averaged T wave (ln µV)** | 0.07 | 0.55 |
| **Amplitude of the Q wave in lead Z (µV)** | 0.08 | 0.48 |
